# Monocyte-Derived Chicken Macrophages Exposed to *Eimeria tenella* Sporozoites Display Reduced Susceptibility to Invasion by *Toxoplasma gondii* Tachyzoite

**DOI:** 10.3390/microorganisms11081999

**Published:** 2023-08-03

**Authors:** Runhui Zhang, Wanpeng Zheng, Arwid Daugschies, Berit Bangoura

**Affiliations:** 1 Key Laboratory of Animal Medicine, Southwest Minzu University of Sichuan Province, Southwest Minzu University, Chengdu 610225, China; 2 Institute of Parasitology, Centre for Infectious Diseases, Leipzig University, 04103 Leipzig, Germany; owen19880508@126.com (W.Z.); daugschies@vetmed.uni-leipzig.de (A.D.); 3 Albrecht-Daniel-Thaer-Institute, 04103 Leipzig, Germany; 4 Department of Veterinary Sciences, University of Wyoming, Laramie, WY 82071, USA; bbangour@uwyo.edu

**Keywords:** chicken, co-infection, *Eimeria tenella*, live cell imaging, macrophages, *Toxoplasma gondii*

## Abstract

Both *Eimeria tenella* and *Toxoplasma gondii* are common apicomplexan parasites in chickens. Host cell invasion by both protozoans includes gliding motility, host cell attachment and active penetration. Chicken macrophages as phagocytic cells participate in the innate host immune response against these two parasites. In this study, primary chicken monocyte-derived macrophages (MM) were infected with both pathogens to investigate mutual and host–parasite interactions. MM cultures were assigned to groups that were infected with *E. tenella*, *T. gondii* or both. In co-infected cultures, MM were first exposed to *E. tenella* sporozoites for 2 h. Afterwards, *T. gondii* tachyzoite infection was performed. Live-cell imaging was carried out to observe cell invasion and survival of *T. gondii* by single parasite tracking over a period of 20 h post infection (hpi). Quantitative analysis for parasite replication was performed by real-time quantitative PCR (qPCR) at 2, 6, 12 and 24 hpi. Overall, the ability of *T. gondii* to penetrate the cell membrane of the potential host cell was reduced, although high motility was displayed. We found that *T. gondii* tachyzoites adhered for more than 4 h to macrophages during early co-infection. qPCR results confirmed that significantly less *T. gondii* entered in *E. tenella*-activated MM at 2 hpi, and a reduced proportion of intracellular *T. gondii* survived and replicated in these cells at 24 hpi. We conclude that *E. tenella* modulates host cell responses to another apicomplexan agent, *T. gondii,* reducing active invasion and multiplication in chicken primary macrophages.

## 1. Introduction

Apicomplexan protozoa are obligate intracellular parasites that cause a variety of diseases in animals and humans [1,2]. *Toxoplasma gondii* infects almost all euthermic animal species and may also infect humans. About one third of the global human population are supposedly infected by this zoonotic pathogen [3]. Chickens are considered to be an important reservoir of *T. gondii*. In particular, free-ranging and back-yard chickens have been reported to show high seroprevalence rates for *T. gondii* [4]. In contrast, coccidiosis caused by *Eimeria* species is a widely distributed major parasitic disease of poultry. One of the most virulent species in chickens is *E. tenella*.

In chickens, macrophages play a crucial role in the identification and phagocytosis of pathogens including protozoan; thus, they serve as a first line of innate immune defense [5]. Upon infection, some apicomplexan parasites are able to establish the specific parasitophorous vacuole (PV) in the host cells, including immune cells, in order to protect themselves. *T. gondii* tachyzoites are able to replicate in chicken blood monocyte-derived macrophages [6]. A previous study showed that *T. gondii* tachyzoites were capable to actively invade macrophages, which occurred even faster than phagocytosis displayed by a macrophage [7]. For *Eimeria*, sporozoites have been found to be situated mainly within or next to the lamina propria, which was infiltrated with macrophages in response to the *E. tenella* infection in naïve chickens [8]. Sporozoite-bearing macrophages are able to transport *Eimeria acervulina* sporozoites to the proper site of the intestinal mucosa [9]. However, *E. tenella* survival and development is rather poor in cultured chicken macrophages [10].

Concomitant infections by protozoan parasites and other microorganisms attract increasing attention in animals and humans both under natural conditions and in experimental in vivo and in vitro studies. For instance, probiotic bacteria reduced oocyst excretion of *E. acervulina* in chicken [11] as well as in vitro invasion of *E. tenella* into Madin-Darby bovine kidney (MDBK) cells [12]. Host immune responses—and consequently, clinical signs of disease—are modulated in co-infected animals. In general, the immune response is dominated by Th1 during protozoan infection, whereas Th2 response is typical for helminth infection [13]. Regarding interaction during co-infection by protozoa, it has been reported that no competitive effects exist in mixed *Eimeria* spp. infection [14]. However, *T. gondii* replication was increased during co-infection with *Trypanosoma lewisi* in rats [15]. In contrast, *T. gondii* supports *Plasmodium berghei* replication in a rat model [16]. Natural co-occurrence of *T. gondii* and *Eimeria* in the same host has been reported. For example, both parasites were found concurrently in the blood and organs of a heavily diseased sparrow [17]. Furthermore, wild rabbits tested seropositive for both *T. gondii* and *Eimeria stiedae* infection in Scotland [18].

In spite of high seroprevalence rates reported for both *T. gondii* and *E. tenella* in chickens, to date, little is known about the mutual interplay between these two parasites, particularly during host cell invasion. Experimental in vivo and ex vivo co-infection models in chicken macrophages were established recently; thus, suitable tools are now available for further co-infection studies [19,20]. Results demonstrated that the mutual interaction during co-infection modulated both parasite replication as well as the host immune response.

In recent years, live-cell imaging has been applied broadly to investigate host–pathogen interaction in macrophages [21,22,23]. In the current study, we used live-cell imaging of *T. gondii* and/or *E. tenella* infected cells to monitor parasite invasion and survival at the single cell-parasite level in both mono-infected and co-infected cultures. The study focuses on the period of early parasite invasion in monocyte-derived chicken macrophages. A previous study showed that asexual stages of *T. gondii*, *E. acervulina* and *E. tenella* displayed ultrastructural similarities [24]. In addition, macrophage phagocytosis was distinctly altered during in vitro co-infection by *T. gondii* and *E. tenella* in our recent study [25]. Thus, we are particularly interested in studying whether the capacity of *T. gondii* to invade and survive in macrophages is affected by host-specific *E. tenella* infection. Parasite tracking was performed to determine the motility of *T. gondii* during the invasion phase following co-infection by *E. tenella*. The lifespan of individual *T. gondii* was also monitored by live cell imaging.

## 2. Materials and Methods

### 2.1. Parasites and Host Cells

Tachyzoites of the *T. gondii* RH-green fluorescent protein (GFP) strain (kindly provided by Professor Dominique Soldati-Favre, University of Geneva Medical School, Geneva, Switzerland) were maintained at 37 °C with 5% CO_2_ in human foreskin fibroblast (HFF) cells. Free tachyzoites were collected from the culture medium for infection. The sporozoites of *E. tenella* Houghton-yellow fluorescent protein (YFP) strain (kindly provided by Prof. Dr. Suo, China Agricultural University, Beijing, China) were obtained from oocysts by excystation following an established protocol [26].

The animal experiments performed to collect chicken blood samples were approved by the responsible authorities (Landesdirektion Sachsen, Chemnitz, Germany, trial registration number V13/10). Chicken peripheral blood mononuclear cells (PBMCs) were isolated from heparinized whole blood of adult chickens according to the established protocols with slight modifications kindly provided by Dr. Braukmann, Friedrich-Loeffler-Institute Jena, Jena, Germany. Briefly, PBMCs were separated from blood by centrifugation (250× *g*, 45 min) with Biocoll (density 1.077 g/mL; Biochrom AG, Berlin, Germany). Isolated PBMCs were resuspended and washed in 5 mL PBS (centrifugation 350× *g*, 30 min) following pre-warmed 5 mL RPMI-1640 medium (Sigma, Taufkirchen, Germany) (centrifugation 350× *g*, 20 min). Afterwards, 5 × 10^6^ PBMCs/well were resuspended in 24-well-plates in RPMI-1640 with 5% chicken serum and 5% fetal bovine serum, penicillin (100 U/mL, PAA), streptomycin (0.1 mg/mL, PAA), and amphotericin B (0.0025 mg/mL, PAA). After 72 h incubation (41 °C, 5% CO_2_) PMBCs were trypsinized by Biotase^®^ (Biochrom, Berlin, Germany) at 37 °C for 30 min. Detached monocyte-derived macrophages (MM) were counted under the microscope. An amount of 10^3^ MM were seeded in the microscope imaging chamber (micro-insert 4 well, Ibidi, Martinsried, Germany) for 24 h (41 °C, 5% CO_2_) for live cell imaging. For parasite quantification by qPCR, approximately 10^5^ MM were seeded to 24-well-plates for 24 h (41 °C, 5% CO_2_). The MM were purified by rinsing off non-adherent cells once at 24 h and twice before infection.

### 2.2. Infection

**Figure 1:**
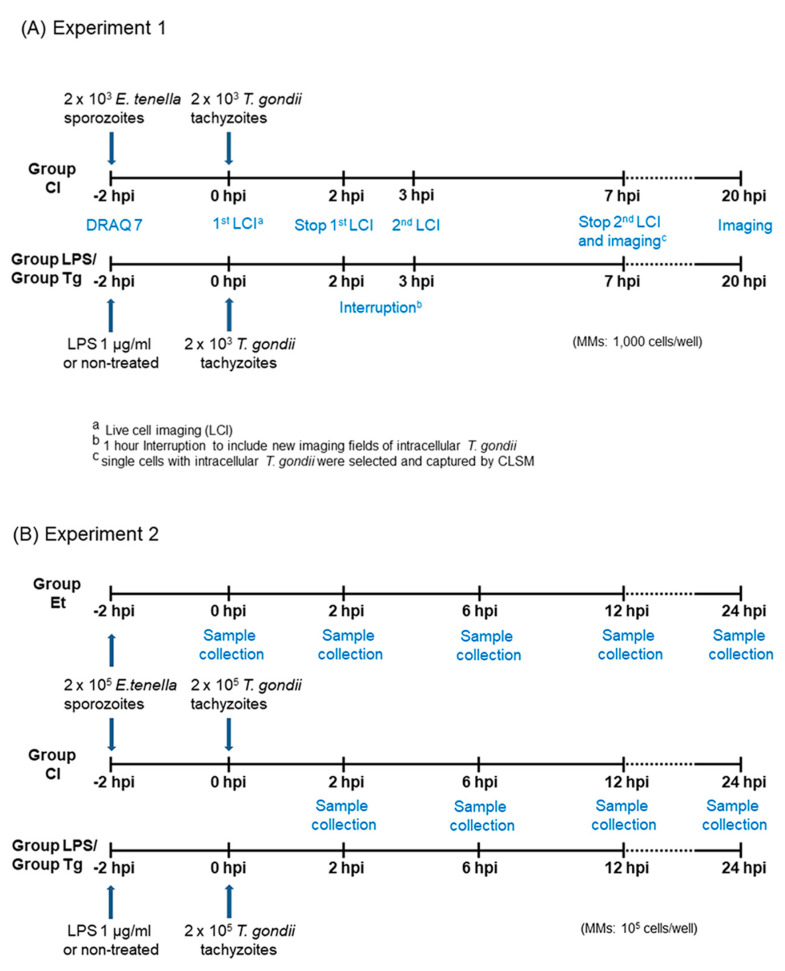
Schematic representation of infection and imaging. (**A**) Experiment 1 (live cell imaging). Images of group NC were collected at the same time points as in groups CI, LPS and Tg; (**B**) Experiment 2 (parasite quantification by qPCR). Samples of group NC were collected at the same time point as in groups CI, LPS and Tg.

Two experiments were conducted in this study (Figure 1): Experiment 1 was designed to visualize parasite invasion; Experiment 2 was performed with increased MM population at the same infection ratio to quantify parasites by qPCR for the different infection groups.

Study design for experiment 1 (Figure 1A):

For imaging, cell cultures were assigned to four groups (n = 2 per group). In the co-infected group (CI), MM were exposed to 2 × 10^3^ *E. tenella* sporozoites for 2 h before *T. gondii* infection (−2 hpi). Group LPS cultures served as positive controls and were stimulated at the same time with 1 μg/mL lipopolysaccharide (LPS). Cultures of group Tg (mono-infection with *T. gondii*) and group NC (negative control) were not exposed to LPS stimulation. At 0 hpi, cultures of groups CI, LPS, and Tg were infected with 2 × 10^3^ *T. gondii* tachyzoites per well; group NC remained uninfected. All cultures were observed until 20 hpi. The whole experiment was repeated once.

Study design for experiment 2 (Figure 1B):

For quantification by qPCR, cell cultures kept in 24−well plates were assigned to five groups (n = 5 cultures/group). At −2 hpi, MM of group CI (co-infection) and group Et (single infection with *E. tenella*) were exposed to 2 × 10^5^ *E. tenella* sporozoites per culture. Group LPS cultures were pretreated with 1 μg/mL LPS at the same time. Group Tg was infected with 2 × 10^5^ *T. gondii* tachyzoites per well at 0 hpi while group NC served as the untreated, uninfected negative control. The cultures were maintained until 24 hpi.

### 2.3. Live-Cell Imaging of T. gondii in MM

DRAQ7 dye was used to assess the viability of parasites and macrophages in each group. Prior to imaging, 3 μL DRAQ7 dye (Biostatus, Leicestershire, UK), a nuclear stain selective for dead cells, was added to all cell cultures immediately after exposure to *E. tenella* or LPS treatment (−2 hpi). The viability of parasites and cells were controlled by fluorescent microscopy prior to *T. gondii* infection at 0 hpi. Cells were viewed for fluorescence by CLSM (TCS-SP8, Leica, Bensheim, Germany) using 4 channels at 488 nm, 514 nm, 633 nm laser line and wide field. Basic imaging parameters were 40× objective, 10× ocular (NA 0.90), 1024× 1024 dpi, 6 Z-stacks (4 μm). To avoid crosstalk between channels, images were collected in a line sequential mode.

Incubation conditions (41 °C, 5% CO_2_ and 99% humidity) were controlled using an incubation chamber (Tokai-Hit, Shizuoka, Japan) over the whole observation period. The timeframe of infection and imaging is shown in Figure 1A. Briefly, after adding DRAQ7, approximately 20 random fields of cells were selected and captured according to groups CI, LPS and Tg from 2 individual wells per group. Image collection for time-lapse imaging of tachyzoite motility was performed at an interval of 10 min per frame until 2 hpi. Image acquisition was interrupted from 2 hpi to 3 hpi to add 20 additional fields per group, which contained intracellular *T. gondii* in group CI and LPS. All fields were observed for a further 4 h until 7 hpi at an interval of 30 min per frame. Subsequently, at least 100 cells with live intracellular *T. gondii* (1–2 tachyzoites/cell) were collected and captured at 7 hpi and 20 hpi by CLSM for groups CI and Tg. For group LPS, only 71 MM with live intracellular *T. gondii* were selected due to a generally low number of *T. gondii*-positive cells observed in this group. Six fields were analyzed randomly for group NC in parallel.

Images of stacks were obtained using LAS X software (Leica, Bensheim, Germany). Stacks were analyzed with Imaris^®^ software version 9.3 (Bitplane, Abingdon, UK) using the functions of spot detection and tracking parasite motility and viability.

### 2.4. Parasite Quantification by Quantitative Real-Time PCR (qPCR)

For all infection groups (Figure 1B), complete cell populations from a subset of cell culture wells were collected at 2, 6, 12, and 24 hpi and additionally at 0 hpi in group Et. DNA was extracted using the QIAamp DNA Mini Kit^®^ (Qiagen, Hilden, Germany) following the manufacturer’s instructions. qPCR was performed using the CFX Connect Real-Time PCR Detection System (Bio-Rad, Hercules, CA, USA). Data represent the mean of three replicates with an acceptable standard deviation of less than 0.5 for Ct values.

*T. gondii* multiplication was analyzed by a probe-based qPCR detecting the 529-bp repeat element [27]. Standard curve was developed by data obtained for gradient 10-fold dilutions of initially 10^7^ tachyzoites. qPCR was conducted in a total volume of 25 µL: 5 µL of sample DNA, 12.5 µL of Master Mix, 3.2 µL of DNase/RNase free water (Gibco™, Life Technologies, NY, USA), 2.5 µL of 2 µM TaqMan probe and 1.125 µM forward and reverse primer (5′-CACAGAAGGGACAGAAGT and 5′-TCGCCTTCATCTACAGTC-3′). The cycling program consisted of 95 °C for 15 min, followed by 40 cycles of 95 °C for 15 s, 60 °C for 1 min and 72 °C for 15 s.

ITS1 fragment quantification was used to assess the replication of *E. tenella* by a SYBR Green-based PCR [28]. The relative copy number of *E. tenella* DNA was implemented by measurement of pSCA-17 plasmid standard dilution, as described previously [29]. qPCR was conducted in a total volume of 20 µL: 2 µL of sample DNA, 10 µL of SYBR Green master mix (Thermo Fisher Scientific, Darmstadt, Germany), 7.2 µL of water, 1.125 µM forward and reverse primer (5′-AACCTGACTGTGCAAGCATC-3′ and 5′-ATCATAGACAGCCGTGCCAG-3′). The cycling program consisted of heating to 95 °C for 5 min, followed by 40 cycles at 95 °C for 30 s, 55 °C for 20 s and 72 °C for 20 s. A subsequent melting curve analysis (95 °C for 1 min, 55 °C for 30 s, 0.5 °C/s) was performed to create the dissociation curve.

### 2.5. Statistical Analysis

Statistical analysis was performed by using Imaris^®^ and GraphPad Prism^®^ (version 8, San Diego, CA, USA) software. The Kolmogorov–Smirnov test was performed to test for normal distribution. Statistical significance was assessed by two-way ANOVA for data with normal distribution and Tukey’s multiple comparisons test for values that did not follow normal distribution.

## 3. Results

### 3.1. Live Cell Imaging of T. gondii in MM

**Figure 2:**
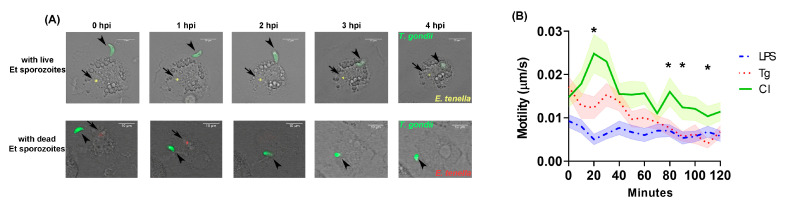
Invasion of *Toxoplasma gondii* within 4 hpi. (**A**) Video microscopy of *T. gondii* invasion in a co-infected cell with an *Eimeria tenella* sporozoite at 0–4 hpi. Upper row represented that *T. gondii* was adherent on the cell that contains a vital *E. tenella* sporozoite. Below row represented that *T. gondii* is adherent on the cell that contains a dead *E. tenella* sporozoite, which was phagocytized and fused by macrophages at 2 hpi. (**B**) Motility (speed) of live *T. gondii* tachyzoites (0–120 min). The motility of *T. gondii* tachyzoites (n = 30) was assessed by imaging every 10 min till 120 min. Each parasite is tracked over 70–100% of all time points considering out-of-range movement or parasite death. Mean value per time point was calculated and presented. LPS: LPS-treated *T. gondii* mono-infection; Tg: *T. gondii* mono-infection; CI: co-infection. * *p* < 0.05, CI compared to Tg; Error bar: standard error of the mean (SEM).

In general, penetration of *T. gondii* tachyzoites into the MM started within 2 hpi after tachyzoites were seeded into cultures. CLSM analysis (Figure 2A and Supplementary Materials Video S1) of group CI cultures showed that *T. gondii* tachyzoites remained loosely adherent to MM for more than 4 h in most cases before they started to actively invade the host cell. During this phase, the distinct helical gliding motility of tachyzoites was observed. This untypical behavior of tachyzoites in group CI was observed regardless of whether intracellular *E. tenella* sporozoites were still alive or dead. In contrast, tachyzoites, which were actively or passively incorporated into MM after attachment, were seen within minutes in the mono-infected group Tg and the LPS-treated group.

Tachyzoites showed the lowest motility in group LPS at most of the captured time points (Figure 2B). In group CI, significantly higher motility of tachyzoites were observed than in group LPS over the period from 10 min to 120 min post infection (*p* < 0.05), with the exception of 70 to 110 min. Comparing between groups CI and Tg, tachyzoites of group CI showed significantly higher motility (*p* < 0.05) during the first 20 min. Average motility values in group CI tended to remain higher than in both groups Tg and LPS with a significant difference (*p* < 0.05) at 80, 90 and 110 min compared to group Tg.

### 3.2. Parasite Quantification

**Figure 3:**
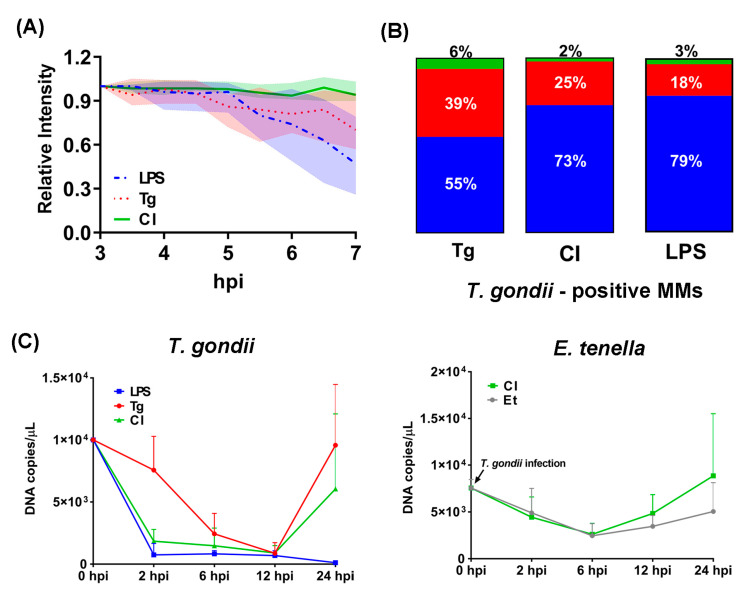
Quantitative analysis of *Toxoplamsa gondii* survival. (**A**) Relative intensity (RI) of intracellular *T. gondii* (n = 27–30) at 3–7 hpi. In order to avoid technical errors such as focus difference per well and potential alteration of fluorescent protein expression by immune cells, all the data are represented as relative intensity (RI). Relative Intensity (RI) = Initial mean intensity of each parasite/Mean intensity of each parasite per time point. RI = 0 when no signal of fluorescence is detected. Error bar: standard error of the mean (SEM). (**B**) The rate of *T. gondii* negative/positive cells at 20 hpi. A total of 100 single cells in group CI and Tg and 71 cells in group LPS (because of low number of survival parasites) were selected and marked randomly at 7 hpi. Each cell with initially viewed 1–2 live intracellular tachyzoites of *T. gondii*/cell were captured at same time point. Marked cells were captured again at 20 hpi and the rate of *T. gondii*–negative/positive cells were calculated. ‘Blue’ represented negative: cells without or with reduced number of live *T. gondii*; ‘red’ represented positive: cells with the same number of initial live *T. gondii*; ‘green’ represented double positive: cell with replicated *T. gondii*. (**C**) Parasite quantities during sequential co-infection in chicken primary macrophages by qPCR. Left line chart represented parasite quantities of *T. gondii*. Right line chart represented parasite quantities of *E. tenella.* Parasite replication is represented as mean value with standard deviation (n = 3–5 per time point). LPS: LPS-treated *T. gondii* mono-infection; Tg: *T. gondii* mono-infection; Et: *E. tenella* mono-infection; CI: co-infection.

In experiment 1, the relative intensity (RI) of green fluorescence of 27 to 30 individual intracellular *T. gondii* tachyzoites that were captured at 3 to 7 hpi by CLSM were anlayzed in all infected groups. RI revealed no significant difference between groups Tg and CI until 4 hpi. Thereafter, RI values remained on a consistently higher level in group CI than in the mono-infected group Tg and in group LPS (Figure 3A). The RI measured in group CI displayed only slight variation from 3 to 7 hpi, whereas values started to decrease in group LPS and Tg following 4.5 hpi or 5 hpi, with a statistically significant difference between groups CI and LPS (*p* < 0.05) at 4.5 hpi.

In general, less than 45% of traced intracellular tachyzoites of *T. gondii* were still alive or replicating in MM at 20 hpi (Figure 3B). At 20 hpi, the proportion of *T. gondii*-positive MM was lower in group CI (27%) than in group Tg (45%), and only slightly higher than in group LPS (21%).

Using qPCR, significantly lower DNA copy numbers (*p* < 0.05) were determined at 2 hpi for *T. gondii* in group CI and group LPS compared to group Tg (Figure 3C, left). The number of DNA copies remained on a low level in the two former groups until 12 hpi, followed by a steep increase until 24 hpi in group CI while values remained significantly lower in group LPS (*p* < 0.05). In group Tg, DNA copy numbers were higher than in the two other groups at 2 hpi, decreased steadily until 12 hpi to a level similar to groups CI and LPS and increased even more distinctly than those recorded for group CI thereafter. No statistical difference was observed between group CI and Tg at this time point (*p* > 0.05). Likewise, no significant difference in DNA copy numbers was detected for *E. tenella* by comparison of groups Et and CI over the entire observation period of 24 hpi (Figure 3C, right).

## 4. Discussion

A recent study by [20] confirmed earlier findings by Long et al. [30], who found that *E. tenella* sporozoites were mostly located within chicken macrophages at 2 hpi. In the current study, most intracellular *E. tenella* sporozoites showed red fluorescence after DRAQ7 staining instead of the expected yellow (YFP) fluorescence within 4 hpi to 6 hpi, indicating their death. This CLSM finding corroborates the qPCR results that demonstrate how the number of *E. tenella* declined to lowest numbers at 6 hpi (Figure 3A, right). Co-infection with *T. gondii* did not exhibit a significant influence on *E. tenella* replication when compared to single *E. tenella* infection. Unfortunately, we could not quantify the *E. tenella* sporozoite numbers via YFP through time lapsing imaging because of a non-ideal YFP expression (about 80%) in the available parasite strain.

Host cell invasion by *T. gondii* tachyzoites usually takes only 15 to 20 s [6]. It is assumed that *T. gondii* tachyzoites and *Eimeria* sporozoites may invade and traverse several host cells by disrupting the host cell membrane [31]. In our time lapsing study, *T. gondii* tachyzoites were either floating free in the culture medium (unattached to macrophages) for hours, or displayed a rapid entry into the macrophages, in both group LPS and group Tg. Interestingly, adherence of vital tachyzoites was prolonged for more than 4 h in the co-infection cultures. It was previously demonstrated that most *T. gondii* tachyzoites remained adherent to murine macrophages that were treated with a phagocytosis inhibitor, Cytochalasin D [32]. Microneme exocytosis is necessary for host–cell entry of both *T. gondii* [13] and *E. tenella* [33]. With these results, we might assume that *E. tenella* infection may potentially hamper the entry of *T. gondii* into host cells by altering recognition of signal receptors or inhibiting phagocytosis.

Rapid invasion and egress are crucial to *T. gondii* survival and successful replication, thereby minimizing the exposure to destructive reactions by innate protection in a generally hostile extracellular environment [34]. During the early stage, up to 2 h after infection, *T. gondii* penetration started. Compared to group Tg, tachyzoites showed significant motility differences (*p* < 0.05) at the beginning (10 min) and after 80 min post infection (80, 90 and 110 min), and significantly lower DNA copy numbers (*p* < 0.05) by qPCR at 2 hpi. In our previous study, only about 10% of applied tachyzoites were able to enter macrophages after *T. gondii* mono-infection at 2 hpi [25]. It may indicate that most tachyzoites stay extracellular or adherent when alive, which is similar with our microscopy observation.

Once inside the host cell, the parasite no longer moves [35]. The intracellular survival of *T. gondii* depends on the route by which the parasite enters the host cell. Although *T. gondii* tachyzoites are phagocytized and internalized through Fc receptor mediation [36], intracellular *T. gondii* can survive and replicate within their PV by blocking the host macrophage’s pathways intended to initiate vacuolar acidification and parasite inactivation [37]. To quantify viable intracellular *T. gondii*, the intensity of GFP signal expression by the parasite was monitored in this study. It appeared from our observations that according to RI values, *T. gondii* showed better tolerance and survival at the early stage of infection (until 7 hpi) if MM were previously exposed to *E. tenella* (Figure 3A).

Under the conditions of our experimental design (two *T. gondii* tachyzoites per cell), most infected host cells contained only one parasite at 7 hpi in all infected groups. By CLSM, only slightly high *T. gondii*-positive cells were monitored in group CI compared to group LPS. However, a significantly high number of total DNA copies of *T. gondii* was detected in group CI compared to group LPS. Therefore, we conclude that the single cell observation by CLSM may not be completely comparable with quantification of DNA copies in cell cultures due to the different amounts of cells considered. Nonetheless, results obtained with both methods indicated reduced growth of *T. gondii* in chicken macrophages in a co-infection setting with *E. tenella* compared to mono-infection.

Macrophages are not only professional phagocytes, but also secrete cytokines in response to parasite infection. This innate immune response can be triggered in chicken macrophages by exposure to LPS [38]. A previous study showed that *T. gondii* blocked LPS-induced production of IL-12 and TNF-alpha in murine bone-marrow-derived macrophages [39]. For *E. tenella*, it is known that IL-1β and iNOS expression are significantly enhanced in chicken HTC macrophages by merozoites at 2 hpi [40]. A recent in vitro study demonstrated down-regulation of IL-12 and iNOS in chicken macrophages during simultaneous co-infection by *T. gondii* and *E. tenella* [20]. From previous data, as well as the current data, it appears that the modulation of innate immunity in chickens differs during mono- and co-infection, which includes cytokine production and macrophage phagocytosis. However, data on concurrent infections with *E. tenella* and *T. gondii* are still scarce, although both are considered to be common pathogens in poultry and consequently deserve more attention.

## 5. Conclusions

In summary, life cell imaging by CLSM proved to be a useful tool to evaluate chicken macrophage invasion and/or phagocytosis during mono- and co-infection with two different apicomplexan parasites. It was demonstrated that the mechanisms of *T. gondii* invasion and survival appear to be altered in *E. tenella*-exposed macrophages. Further studies into macrophage signaling pathways, particularly modulation of macrophage polarization (such as cytokines, functional genes), combined with image analysis and live cell imaging, will help to better understand the function and modulation of the innate immune response during apicomplexan invasion.

## Data Availability

The data presented in this study are available on request from the corresponding author.

## Supplementary Materials

The following supporting information can be downloaded at: https://www.mdpi.com/article/10.3390/microorganisms11081999/s1, Video S1: Video microscopy of *Toxoplasma gondii* invasion in a co-infected cell with an *Eimeria tenella* sporozoite at 0–4.5 hpi.
