# Supplementary material for: Monocyte-Derived Chicken Macrophages Exposed to Eimeria tenella Sporozoites Display Reduced Susceptibility to Invasion by Toxoplasma gondii Tachyzoite
Source: Microorganisms. 2023 Aug 3;11(8):1999. doi: 10.3390/microorganisms11081999 (PMC10460027; doi:10.3390/microorganisms11081999)
Supplement: Supplementary file 1 [file microorganisms-11-01999-s001.zip › microorganisms-2497733-supplementary.pdf]

Supplementary Material

Supplementary Video 1 Video microscopy of *T. gondii* invasion in a co-infected cell with an *E. tenella* sporozoite at 0-4.5 hpi. *T. gondii* is adherent on the cell which contains a vital *E. tenella* sporozoite. Yellow fluorescence: *E. tenella* sporozoites; green fluorescence: *T. gondii* tachyzoites; wide field: single macrophage.
